# shRNA-mediated gene silencing of HDAC11 empowers CAR-T cells against prostate cancer

**DOI:** 10.3389/fimmu.2024.1369406

**Published:** 2024-05-21

**Authors:** Hongmei Zhang, Jie Yao, Iqra Ajmal, Muhammad Asad Farooq, Wenzheng Jiang

**Affiliations:** Shanghai Key Laboratory of Regulatory Biology, School of Life Sciences, East China Normal University, Shanghai, China

**Keywords:** chimeric antigen receptor (CAR), immunotherapy, histone deacetylases (HDAC), solid tumor, prostate cancer

## Abstract

Epigenetic mechanisms are involved in several cellular functions, and their role in the immune system is of prime importance. Histone deacetylases (HDACs) are an important set of enzymes that regulate and catalyze the deacetylation process. HDACs have been proven beneficial targets for improving the efficacy of immunotherapies. HDAC11 is an enzyme involved in the negative regulation of T cell functions. Here, we investigated the potential of HDAC11 downregulation using RNA interference in CAR-T cells to improve immunotherapeutic outcomes against prostate cancer. We designed and tested four distinct short hairpin RNA (shRNA) sequences targeting HDAC11 to identify the most effective one for subsequent analyses. HDAC11-deficient CAR-T cells (shD-NKG2D-CAR-T) displayed better cytotoxicity than wild-type CAR-T cells against prostate cancer cell lines. This effect was attributed to enhanced activation, degranulation, and cytokine release ability of shD-NKG2D-CAR-T when co-cultured with prostate cancer cell lines. Our findings reveal that HDAC11 interference significantly enhances CAR-T cell proliferation, diminishes exhaustion markers PD-1 and TIM3, and promotes the formation of T central memory T_CM_ populations. Further exploration into the underlying molecular mechanisms reveals increased expression of transcription factor Eomes, providing insight into the regulation of CAR-T cell differentiation. Finally, the shD-NKG2D-CAR-T cells provided efficient tumor control leading to improved survival of tumor-bearing mice *in vivo* as compared to their wild-type counterparts. The current study highlights the potential of HDAC11 downregulation in improving CAR-T cell therapy. The study will pave the way for further investigations focused on understanding and exploiting epigenetic mechanisms for immunotherapeutic outcomes.

## 1 Introduction

Chimeric antigen receptor (CAR)-T cell therapy has emerged as a promising strategy to combat cancer in several animal and human clinical trials (1, 2). CAR-T cell therapy utilizes gene editing of T cells to enhance tumor-specific lysis and has gained enormous success in hematological malignancies (3, 4). Nonetheless, parallel success is still awaited in solid tumors due to a number of factors. That is why, targeting solid tumors with different variants of CAR-T therapy is a prerequisite.

Natural killer group 2 D (NKG2D) receptors can specifically target most cancers without affecting healthy cells, thus presenting a valid chimeric antigen receptor candidate (5). Effector immune cells are triggered to become more active, expand, and to release inflammatory mediators as a consequence of the interaction between the NKG2D ligand (NKG2DL) and NKG2D receptor, which normally results in the elimination of target cells. In our previous findings, NKG2D-based CAR-T cell therapy has shown some promising results in prostate and pancreatic cancer models both *in vitro* and *in vivo* (6–8). Targeting NKG2DL is a promising strategy to treat solid cancers, because of its target specificity and safety profile (7, 9). It was reported previously by our group that NKG2D can be used as a target in prostate cancer immunotherapy. Co-expression of IL-7 in NKG2D-targeted CAR-T cells further enhanced their function against prostate cancer (6). Although NKG2D-targeted CAR-T cells has yielded promising results in preclinical trials, the similar success has not yet translated at clinical levels due to several reasons (5).

The function of CAR-T cells is tightly controlled by the genes they express, and the gene expression is in turn regulated by epigenetic mechanisms. Histones can experience various types of posttranslational modifications (PTMs), such as acetylation, methylation, and others. The process of acetylation is controlled by acetyl transferases and histone deacetylases (HDACs) referred to as ‘writers’ and ‘erasers’ respectively in epigenetic terms (10). Acetylated histones are erased by HDACs resulting in diminished effector and memory functions of T lymphocytes (11). Histone deacetylase 11 (HDAC11) is the most recently discovered enzyme in HDAC class and has been reported to modulate immune cell functions at epigenetic levels. For example, it plays a role in the expansion of myeloid-derived suppressor cells (MDSCs). Downregulation of the HDAC11 gene can boost the effector as well as memory function of T cells as described in previously published data (12). Knockdown of HDAC11 in mice increased T cell lymphoma aggressiveness as compared to wild-type (13, 14). Due to the nonredundant nature of epigenetic enzymes, modulating the gene expression of even a single epigenetic enzyme can change the overall expression profile of CAR-T cells (11). That is why, we speculated that interfering HDAC11 in CAR-T cells could be an excellent target to augment immunotherapeutic efficacy. Previous reports have showed that the efficacy of NKG2D-based CAR-T cell therapy against ovarian cancer and leukemia was enhanced by upregulating the expression of NKG2DL in target cells with HDAC inhibitor VPA (15, 16). Our study presents a unique strategy where HDAC11 downregulation was employed in effector (CAR-T) cells via RNA interference (RNAi) technology. This modification led to improved CAR-T cell therapy efficacy against prostate cancer cells both *in vitro* and *in vivo*.

## 2 Materials and methods

### 2.1 Cell lines and culture conditions

The human prostate cancer cell lines PC-3 (CRL-1435), DU-145 (HTB-81), and the human embryonic kidney cell line HEK293T cells (CRL-3216) were obtained from the American Type Culture Collection (ATCC). PC-3, DU-145, and HEK-293T cells were maintained in Dulbecco’s modified eagle’s medium (DMEM, Gibco Laboratories, Grand Island, NY), along with 10% v/v heat-inactivated fetal bovine serum (FBS, Gibco Laboratories) and 1% v/v penicillin/streptomycin in a humidified incubator at 37°C with 5% CO_2_. Firefly luciferase lentivirus was transduced into the parental PC-3 cell line to express luciferase and termed the PC-3 luciferase cell line. All cell lines were screened regularly to confirm that they were mycoplasma-free.

### 2.2 shRNA design and construction

shRNA was designed by using the Homo sapiens Histone deacetylase HDAC11 mRNA sequence as a template strand (Accession # NM_024827.3), and an online shRNA designing tool was used to obtain the best target interference sequences (http://rnaidesigner.thermofisher.com/rnaiexpress/sort.do) as explained in our previous study (7). Four interference sequences were selected according to their GC contents (30-50%), and then the double-stranded shRNA was designed, which includes these interference sequences along with a loop and 6 T-tail sequences. EcoRI restriction digestion enzyme sequence was also added to identify successful insertion. XhoI and HpaI enzyme sequences were used for sticky and blunt end production, respectively (**Supplementary Table S1**). pLL3.7 lentiviral plasmid was linearized using HpaI and XhoI endonucleases, and a double-stranded shRNA sequence was cloned into it using the most efficient T4 DNA ligase according to the manufacturer’s instruction (New England BioLabs, China). All constructs were verified using specific (EcoRI) restriction digestion electrophoresis and DNA sequencing methods.

### 2.3 CAR design and construction

A peptide encoding NKG2D extracellular domain, followed by CD8α hinge and transmembrane region, 4-1BB co-stimulatory and CD3ζ signaling domain, was already constructed in our laboratory, as explained in our previous studies (6, 7). A novel CAR with downregulated HDAC11 gene expression was developed by incorporating the HDAC11-shRNA sequence into the NKG2D-CAR construct and naming it sh-NKG2D-CAR. A random sequence was also incorporated into NKG2D-CAR to construct the negative control (NC-NKG2D-CAR) lentiviral plasmid.

### 2.4 Lentiviral packaging

The constructed CAR plasmids, along with psPAX2 and pMD2.G packaging plasmids, were co-transfected into HEK293T cells using Polyethyleneimine (PEI) transfection reagent system (Polysciences, Warrington, PA, USA) as described previously (6–8). After transfection, the supernatant containing the lentivirus was collected and concentrated 100 times by ultracentrifugation. The lentiviral titration was measured using the flow cytometry method, based on GFP or NKG2D expression as transducing units per mL (TU/mL).

### 2.5 CAR-T cell preparation

PBMCs were isolated from the peripheral blood of healthy donors (Shanghai Blood Bank, Shanghai, China) by using the Ficoll-Paque density gradient centrifugation method (HyClone, Logan, UT, USA) and activated with 1µg/mL anti-CD3/CD28 antibodies (Miltenyi, Bergisch Gladbach, Germany) for 2 days. Primary T cells were then cultured in X-VIVO™ medium (Lonza, Switzerland), supplemented with 1% penicillin/streptomycin, and 200IU/mL of human recombinant interleukin-2 (IL-2, Seaform Biotech., Beijing, China) at 37°C in 5% CO_2_ incubator. The respective lentivirus was transduced into T cells at an MOI of 10 to construct CAR-T cells. Transduction efficiency was measured by flow cytometry.

### 2.6 Cytotoxicity assays

#### 2.6.1 LDH release assay

To detect cytotoxicity, a lactate dehydrogenase (LDH) release assay was performed according to the manufacturer’s instructions (HY-K0301, MedChem express, NJ, USA). Target and effector cells were co-cultured at various effector-to-target (E: T) ratios (10: 1, 5: 1, and 1: 1) for 12 hours. LDH lysis and stop solution were added, and the absorbance was measured at 490nm using a microplate reader (Thermo Fischer Scientific, USA).

#### 2.6.2 Annexin V/PI-based flow cytometry assay

Cytotoxicity was also measured by flow cytometry, as described in our previous study (6, 7). Target cells were labeled with CFSE dye according to the manufacturer’s instruction (65-0850-84, eBioscience™, Waltham, MA, USA) and subsequently co-cultured with effector T cells at various E: T ratios (10: 1, 5: 1, and 1: 1) overnight. Cells were collected and stained with Annexin V antibody (640920, Biolegend) and PI for 15 minutes in the dark. Apoptotic cells were measured using the double-fluorescence via flow cytometry.

### 2.7 Proliferation assay

Transduced T cells were stained with CFSE dye (65-0850-84, eBioscience™, Waltham, MA, USA) and cultured in the presence or absence of target cells. After 5 days, CFSE dilution was measured by flow cytometry in the FITC channel.

### 2.8 Flow cytometry analysis

Flow cytometry was conducted following standard protocols. Antibodies such as BV421-conjugated anti-human CD3 (317344, Biolegend), APC-conjugated anti-human NKG2D (558071, BD Biosciences), APC-conjugated anti-human CD69 (310910, Biolegend), PE/Cy7-conjugated anti-human CD107a (328618, Biolegend), PE-conjugated anti-human PD-1 (329906, Biolegend), APC-conjugated anti-human TIM3 (345012, Biolegend), APC-conjugated anti-human CD4 (555349, BD Biosciences), PE-conjugated anti-human CD8 (12-0088-42, eBioscience™), PE-conjugated anti-human CD62L (12-0621-82, eBioscience™), and APC/Cy7-conjugated anti-human CD45RA (304127, Biolegend), were directly applied for surface staining.

For intracellular staining, cells were fixed and permeabilized, followed by staining with intracellular antibodies such as APC-conjugated anti-human IFN-γ (502512) and PE/Cy7-conjugated anti-human GzmB (372214) antibodies obtained from Biolegend. The nuclear membrane was permeabilized using a Cytofix Perm kit (BD Biosciences), and Eomes was detected by PE-conjugated anti-human Eomes antibody (157705, Biolegend). Forward vs side, scatter gating strategy was employed, as explained in **Supplementary Figure S4**.

### 2.9 Quantitative PCR analysis

Transduced T cells were collected, and total mRNAs were isolated by the TRizol reagent method (Invitrogen, Shanghai, China) and reverse transcribed into cDNA by using a Hifair 1^st^ strand cDNA synthesis kit (Yeasen Biotech, Shanghai, China). 100ng cDNAs were used further to perform the qPCR analysis. HDAC11 primers were synthesized from Primer Bank with a sequence: forward 5’-GGGTGCCCATCCTTATGGTG-3’ and reverse 5’-CAGCGGTGTGTCTGAGTTCT-3’. The SYBR Premix Ex TaqII qPCR master mix (Takara Bio, San Jose, CA, USA) was used to conduct the qPCR according to its manufacturer’s instructions. GAPDH served as a reference, and the 2^-ΔΔCT^ method was used to analyze the data.

### 2.10 Western blot analysis

Total protein was isolated from transduced CAR-T cells using RIPA lysis buffer, and the concentration was measured using a BCA assay. An equal protein concentration from each sample was loaded on SDS-PAGE gel, and the electrophoresis was performed. Samples were transferred to PVDF nitrocellulose membrane and blocked by skimmed milk. The primary antibody of HDAC11 (ab246512, 1:1000, Abcam, Cambridge, UK) and secondary antibody (Horseradish peroxidase-conjugated goat anti-rabbit IgG antibody) was used to stain the samples. The image was obtained using ImmunoStar Zeta and ImageQuant (GE Healthcare).

### 2.11 *In vivo* xenograft model

Female NOD/SCID/y-chain-/- (NSG) mice, aged eight weeks, were used to establish prostate cancer xenografts. Initially, 5×10^6^ PC-3 luciferase cells were subcutaneously injected into the right flank of mouse. After three weeks, tumor was established and reached up to 100-300mm^3^. Mice were divided randomly in three groups (n=7/group). Mock-T cells (1×10^7^), NKG2D-CAR-T cells (1×10^7^) and shD-NKG2D-CAR-T cells (1×10^7^) were injected intravenously in tumor bearing mice via tail vein 48 hours after the lentivirus infection. IVIS live imaging was performed weekly and tumor volume was assessed using vernier calipers. On day 50, mice blood was extracted using the retro-orbital method, and functional markers were analyzed using flow cytometry. Mice were further sacrificed and tumor tissues were excised. After preparing single cell suspension, tumor-infiltrating cells were detected using BV421-conjugated anti-human CD3 antibody via flow cytometry.

### 2.12 Statistical analysis

GraphPad Prism (version 9) was utilized for graph generation and statistical analysis. Data is represented with mean and standard deviation ( ± SD). One-way ANOVA and two-way ANOVA tests were used according to the experimental requirement. Statistical significance levels are indicated by p-value (*p<0.05, **p<0.01, ***p<0.001, ****p<0.0001), and ns denotes not significant.

## 3 Results

### 3.1 HDAC11 shRNA design and construction

**Figure 1:**
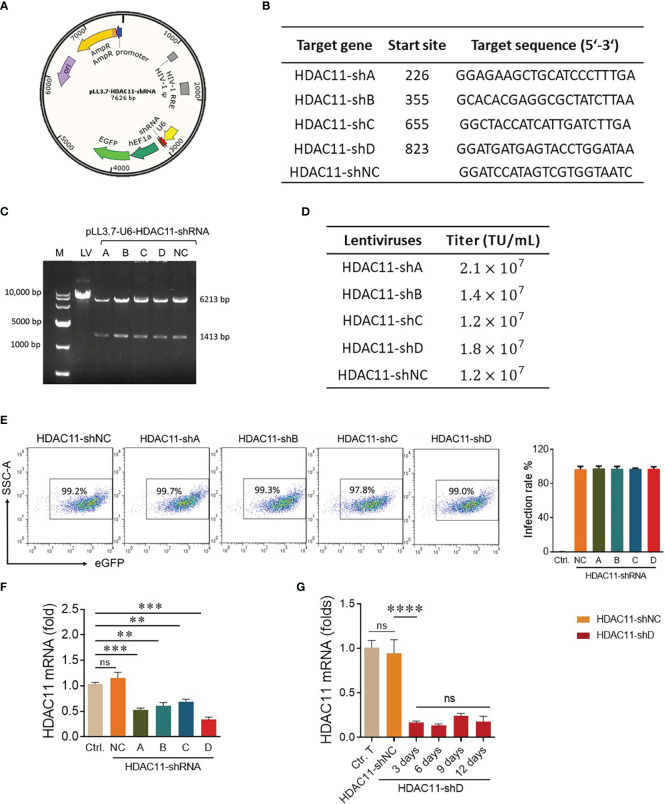
Construction and validation of HDAC11-shRNAs. **(A)** The schematic representation of dual-promoter lentiviral plasmid contains the HDAC11 targeting shRNA sequence and eGFP sequence simultaneously. U6 promoter drives HDAC11-shRNA expression, while EF1a promoter controls eGFP expression. **(B)** Selected nucleotide sequences for HDAC11-shRNA construction. Four nucleotide fragments of the HDAC11 gene were selected and termed HDAC11-(shA, shB, shC, and shD), whereas a scrambled sequence was used as a negative control (HDAC1-shNC). **(C)** Identification of successful construction of pLL3.7 lentiviral plasmids containing HDAC11-shRNAs via restriction digestion electrophoresis method. Lane 1 indicated the 10,000 bp DNA marker. The digested plasmids, including pLL3.7 dual-promoter lentivirus plasmid (lane 2), pLL3.7 containing HDAC11-shRNA variants A-D (lane 3-6), and the negative control (lane 7), were visualized on 1% agarose gel after EcoRI restriction enzyme digestion. **(D)** The transduction efficiency of pLL3.7 lentiviral vectors containing HDAC11-shRNAs was determined in HEK293T cells by flow cytometry. GFP positive rate was measured at different lentivirus concentrations, and titer was calculated using 0.6µL lentiviruses concentration. **(E)** Transfection efficiency in T cells. Primary T cells were infected with each lentivirus at an MOI of 10 for 48 hours, and eGFP positive rate was measured using flow cytometry. Data is presented in the FACS plot (left panel), and the percentage of GFP expression is represented in bar chat (right panel). **(F)** qPCR analysis of HDAC11 knockdown expression in T cells. 2×10^6^ T cells were infected with respective lentiviruses for 48 hours, and mRNA expression was measured using the gene-specific primers. **(G)** T cells containing HDAC11-shD lentivirus were cultured for up to 12 days, and mRNA was extracted at different time points to assess the stability of HDAC11 knockdown via qPCR. The 2^-ΔΔCT^ method was used for data analysis, with GAPDH serving as a reference gene. Statistical significance is indicated by p-value (**p<0.01, ***p<0.001, ****p<0.0001) and ns denotes not significant.

Previously, the deletion of HDAC11 in T cells has led to an increase in overall T cell function (17). That is why we speculated that inhibiting this checkpoint gene in CAR-T cells will boost their efficacy against solid tumors. The pLL3.7 plasmid containing the shRNA sequence is illustrated in **Figure 1A**. Four distinct nucleotide sequences for HDAC11-shRNA were selected with higher GC content (**Figure 1B**) and shRNA oligonucleotides were designed (**Supplementary Table S1**). The interference sequences of HDAC11 were inserted into pLL3.7 lentiviral vectors after vector linearization and subsequent ligation. The successful incorporation of our inserts into the pLL3.7 vector was confirmed by single-enzyme restriction digestion (**Figure 1C**) and sequencing (data not shown). Respective plasmids were termed HDAC11-shA, HDAC11-shB, HDAC11-shC, and HDAC11-shD. A scrambled sequence was also inserted into the cloning vector and was termed HDAC11-shNC.

After successful vector construction, the HEK293T cells were co-transfected with an shRNA plasmid, packaging plasmid, and envelope plasmid to generate lentivirus particles for HDAC11-shA, HDAC11-shB, HDAC11-shC, HDAC11-shD, and HDAC11-shNC. The lentiviruses of newly constructed plasmids displayed dose-dependent transduction efficiency in HEK-293T cells as demonstrated in our FACS data (**Supplementary Figure S1**), and the titer was calculated. Each lentivirus expresses a high amount of titer, as explained in **Figure 1D**. Moving forward, primary human T cells were infected with the experimental cohorts mentioned above and cultured for 48 hours. Later, the cells were collected and analyzed by FACS for GFP-positive cells. The transduction efficiency of experimental cohorts was also high in respective transduced T cells (**Figure 1E**).

To assess interference efficiencies, qPCR was performed in transduced T cells. All four shRNA groups displayed notable downregulation in transduced T cells. Notably, HDAC11-shD exhibited the highest interference efficiency (**Figure 1F**). In HDAC11-shD-transduced T cells, persistent downregulation was recorded till 12 days (**Figure 1G**). The extended period of downregulation highlights the potential long-lasting effect of this shRNA sequence. Overall, our results affirm the effective downregulation of HDAC11 using the selected shRNA sequences, particularly highlighting the superior interference efficiency of HDAC11-shD.

### 3.2 Novel chimeric antigen receptor design and construction

**Figure 2:**
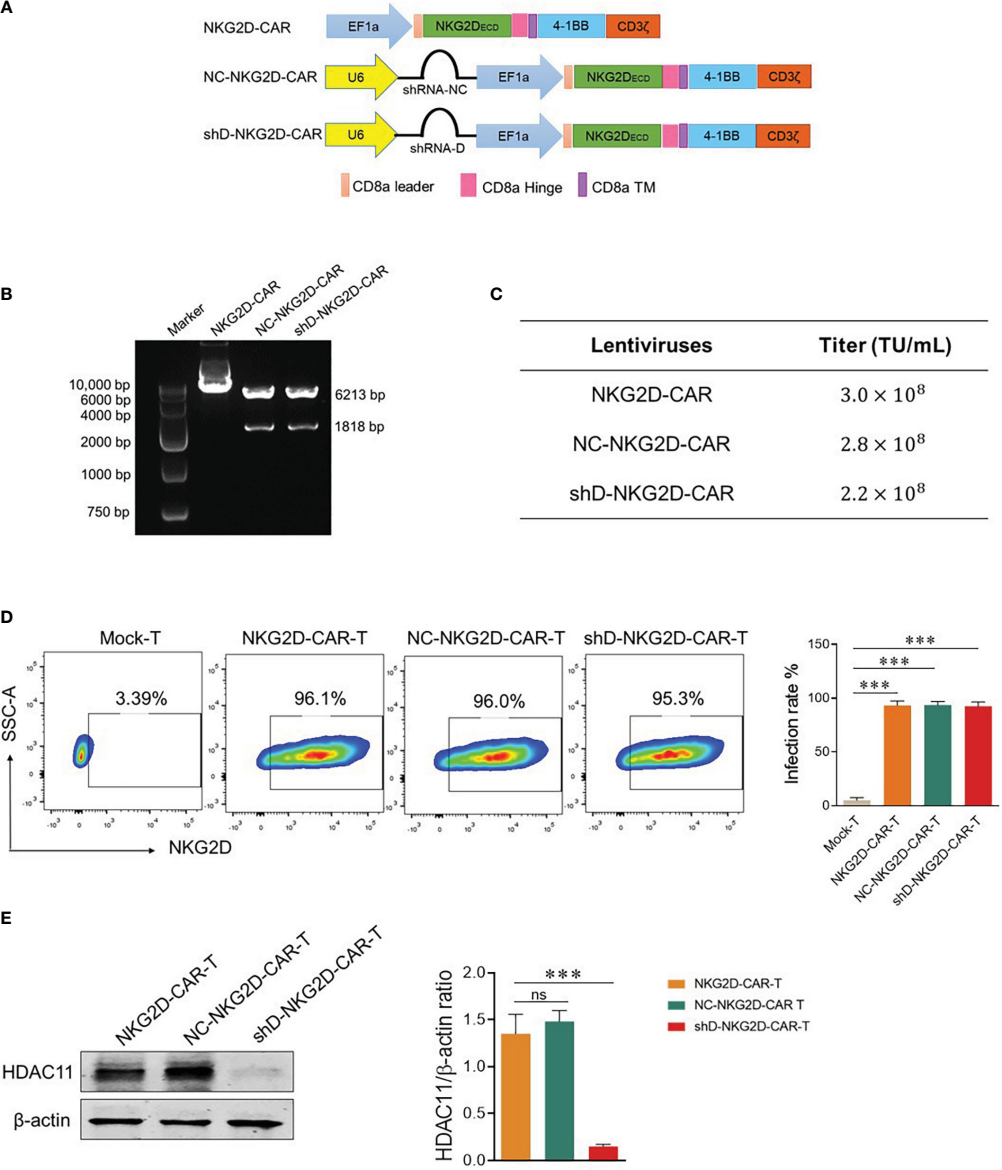
Construction of HDAC11-silenced NKG2D-CAR vector. **(A)** Schematic representation of the two-in-one lentiviral vector containing the NKG2D-CAR and HDAC11-shRNA sequences. **(B)** Confirmation of HDAC11-shRNA sequence ligation into the NKG2D-CAR lentiviral vector was achieved through restriction digestion electrophoresis. Single enzyme restriction digestion with EcoRI produced two bands (6213bp and 1818bp), indicating successful shRNA sequence ligation in the NKG2D-CAR vector. **(C)** HEK293T cells (2×10^5^) were infected with expression, envelope and packaging plasmids using PEI lentiviral packaging system. After 48 hours, cells were stained with APC-conjugated anti-human NKG2D antibody, positive rate was measured using flow cytometry and titer was calculated. **(D)** NKG2D-CAR, NC-NKG2D-CAR, and shD-NKG2D-CAR lentiviruses were transduced into primary T cells at an MOI of 10. After 48 hours, cells were collected and stained with APC-conjugated anti-human NKG2D antibody. Flow cytometry was employed to measure the transduction efficiency. **(E)** HDAC11 knockdown in NKG2D-CAR transduced T cells was confirmed through western blot analysis. β-actin served as the reference protein. The left panel illustrates the blot image, while the right panel presents the relative band intensity of HDAC11 protein compared to β-actin in a bar chart. Statistical significance is indicated by p-value (****p<0.0001), and ns denotes not significant.

Second generation NKG2D-based CAR used in this study is described in (**Figure 2A**) and our previously published data (6, 7). HDAC11-shD and negative control sequences were cut from HDAC11-shD and HDAC11-shNC vectors by restriction digestion and ligated with linearized NKG2D-CAR vector, confirmed via restriction digestion electrophoresis. The resultant NKG2D-CAR vectors containing the HDAC11-shD sequence and the negative control sequence were designated as shD-NKG2D-CAR and NC-NKG2D-CAR, respectively (**Figure 2B**).

Lentiviruses were produced using HEK-293T cells and titer was calculated. Each CAR construct gives a titer of more than 10^8^ TU/mL (**Figure 2C**). T cells were isolated from human PBMCs and were infected with NKG2D-CAR, NC-NKG2D-CAR, and shD-NKG2D-CAR lentiviruses to generate respective CAR-T cells. All three NKG2D-based CAR-T cells were stained with anti-human NKG2D antibody to detect surface expression of NKG2D receptors as a measure of transduction efficiency. All experimental cohorts displayed excellent transduction efficiencies. Comparisons were made with Mock-T cells as a baseline. The comparable transduction efficiency of NKG2D-CAR and shD-NKG2D-CAR signifies that the insertion of the HDAC11 interference sequence didn’t affect the transduction efficiency of second-generation CAR (**Figure 2D**). Western blot analysis indicated that HDAC11 protein expression was significantly downregulated in shD-NKG2D-CAR-T cells (**Figure 2E**). The outcomes of **Figure 2** collectively confirm the effective transduction of NKG2D-CAR-T cells using the HDAC11-shRNA sequence, leading to the successful downregulation and signifying the integration and functionality of the shD sequence within the NKG2D-CAR plasmid.

### 3.3 HDAC11 downregulation improves CAR-T cells cytotoxic aptitude against prostate cancer cell lines *in vitro*

**Figure 3:**
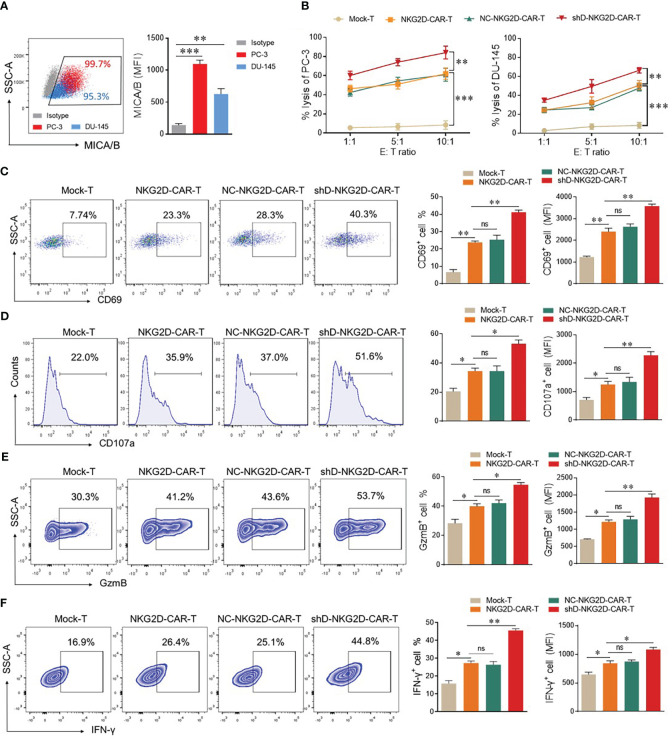
HDAC11 downregulation enhances the anti-tumor function of NKG2D-CAR-T cells. **(A)** Flow cytometry was employed to assess MICA/B expression on PC-3 (depicted in red) and DU-145 cells (depicted in blue). The percentage and mean fluorescent intensity (MFI) were determined using a PE-conjugated anti-human MICA/B antibody. Data is represented in a FACS dot plot (left panel) and bar chart (right panel). **(B)** Cytotoxicity against PC-3 and DU-145 cancer cells was evaluated through an LDH release assay. Effector cells were co-cultured with target cells at various effector-to-target ratios (E: T) for 12 hours, and the resulting percentage lysis was quantified by measuring absorbance at 490nm. **(C–F)** Flow cytometry was employed to profile functional markers in experimental cohorts co-cultured with PC-3 cells at an E: T ratio of 5: 1. After 12 hours, cells were stained with APC-conjugated anti-human CD69 and PE/Cy7-conjugated anti-human CD107a antibodies to measure the positive rate of CD69 **(C)** and CD107a **(D)** respectively. Following 20 hours of co-culture, cells were stained with PE/Cy7-conjugated anti-human GzmB and APC-conjugated anti-human IFN-γ antibodies for analysis of GzmB **(E)** and IFN-γ **(F)** expression. A forward vs. side scatter gating strategy was applied to analyze marker expression under CD3-positive gated cells. Representative FACS data is shown on the left with percentage and MFI presented in bar charts on the right. Statistical significance was determined using a one-way ANOVA test and represented by p-value (*p<0.05, **p<0.01, ***p<0.001), and ns denotes not significant.

The flow cytometry-based expression analysis of NKG2D ligands, MHC class-1 chain-related protein A and B (MICA/B) was conducted in PC-3 and DU-145 cells. Both cell lines exhibited high expression levels (>90%) of MICA/B, rendering them suitable targets for NKG2D-CAR-T cells (**Figure 3A**). Next, we investigated the impact of HDAC11 downregulation on the cytotoxic potential of CAR-T cells, co-cultured with PC-3 and DU-145 cells by lactate dehydrogenase (LDH) release assay. The results revealed an increased cell lysis in target cells co-cultured with HDAC11-deficient CAR-T cells compared to target cells co-cultured with conventional CAR-T cells (**Figure 3B**). The data were further verified in a flow cytometry-based cytotoxicity assay. To assess the killing efficiency, target cells were labeled with CFSE and co-incubated with effector cells at varying effector-to-target (E: T) ratios. The cells were collected and stained with Annexin V and PI to quantify the percentage of apoptotic target cells within the CFSE gate. In NKG2D-CAR transduced T cells, we observed a dose-dependent increase in the killing of target cells when compared with Mock-T cells. No significant difference was noted between NKG2D-CAR-T and NC-NKG2D-CAR-T groups. The shD-NKG2D-CAR-T cells exhibited a substantial increase in the apoptosis rate of target cells (PC-3) at different E: T ratios, showing a significant difference when compared to NKG2D-CAR-T cells (**Supplementary Figure S2**). These findings indicated that HDAC11 downregulation in CAR-T cells enhances their cytotoxic potential against PC-3 and DU-145 prostate cancer cell lines.

To detect the impact of HDAC11 downregulation on activation and degranulation, FACS analysis was carried out for surface expression of CD69 and CD107a. Mock-T cells and CAR-T experimental groups were co-incubated with target cells (E: T ratio of 1: 1) for 12 hours. Subsequently, cells were collected and stained with anti-human CD69 and CD107a antibodies in separate experiments. The data revealed that the shD-NKG2D-CAR-T cells exhibited a significantly higher proportion of CD69^+^ cells (23.3% ± 0.77 vs. 23.3% ± 1.27) and CD107a^+^ cells (35.9% ± 1.98 vs. 51.6% ± 2.40) compared to NKG2D-CAR-T cells (**Figures 3C, D**). A significant difference in the mean fluorescence intensity (MFI) of both groups was also observed. The co-culture of experimental cohorts with DU-145 cell lines also exhibited an increasing trend in CD69 and CD107a expressing cells in HDAC11-downregulated CAR-T cells (**Supplementary Figure S3**). Furthermore, intracellular GzmB (41.2% ± 1.62 vs. 53.7% ± 1.41) and IFN-γ (26.4% ± 1.202 vs. 44.8% ± 0.98) expression were also significantly increased in HDAC11-deficient CAR-T cells (**Figures 3E, F**). These findings highlight that the enhanced cytotoxic effect of HDAC11-silencing CAR-T cells against prostate cancer attributed to the heightened activation, degranulation, and effector molecule expression.

### 3.4 HDAC11 downregulation enhances CAR-T cell proliferation, decreases exhaustion, and promotes memory cell formation *in vitro*

**Figure 4:**
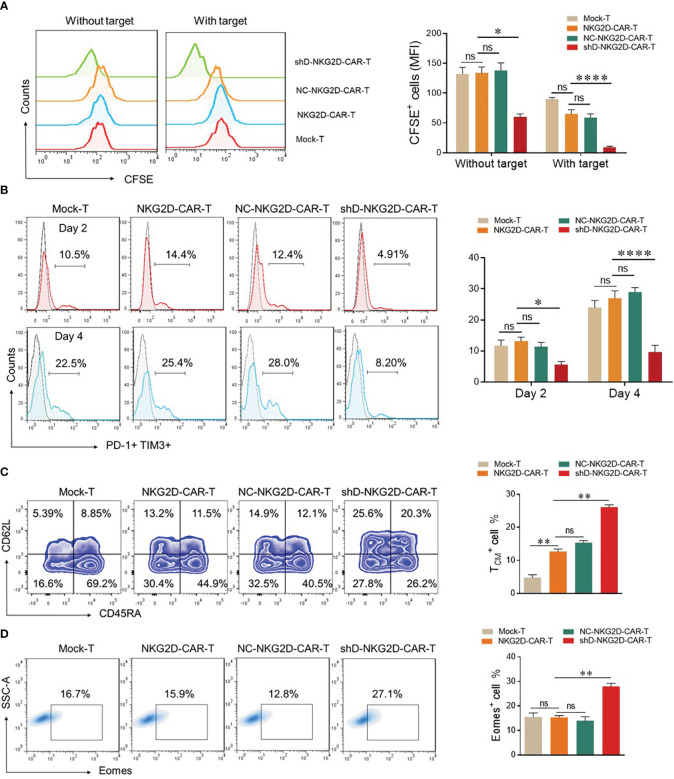
HDAC11 downregulation increases the less differentiated T cell population in NKG2D-CAR-T cells. **(A)** For proliferation assessment, 1×10^6^ cells from each experimental cohort were labeled with 1µL of CFSE. To confirm CFSE labeling, 3×10^5^ cells underwent flow cytometry, and positive cells were detected in the FITC channel. CFSE-labeled cells were then cultured alone or with PC-3 target cells for 5 days at an E: T ratio of 5: 1, and CFSE dilution was measured using the same FITC channel voltage. Data is presented in a FACS histogram on the left side, while the MFI of CFSE illustrated in a bar chart on the right. **(B)** To assess exhaustion, experimental cohorts were co-cultured with PC-3 cells at an E: T of 5: 1. After 2 and 4 days of co-culture, cells were stained with PE-conjugated anti-human PD-1 and APC-conjugated anti-human TIM3 antibodies, and flow cytometry was performed. PD-1 positive cells were detected in the TIM3 positive cell gate to determine double-positive cells, employing a forward vs. side scatter plot. **(C, D)** For T cell differentiation and Eomes detection, experimental cohorts were cultured with PC-3 cells at an E: T of 10: 1. **(C)** After 7 days, cells were stained with BV421-conjugated anti-human CD3, APC/Cy7-conjugated anti-human CD45RA, and PE-conjugated anti-human CD62L antibodies and analyzed by flow cytometry. CD62L and CD45RA double-fluorescence plots in the CD3-positive cell gate were used to detect T cell differentiation populations with CD62L^+^ CD45RA^+^ cells indicating T_CM_. Representative FACS files are displayed on the left side, while the T_CM_ population percentage is illustrated in a bar chart on the right. **(D)** Cells were collected, washed, fixed, and permeabilized with TF perm buffer for 1 hour. After permeabilization, cells were stained with PE-conjugated anti-human Eomes antibody and subjected to flow cytometry. Forward vs. side, scatter gating strategy was applied in all the experiments, and statistics were performed using one-way ANOVA. Statistical significance is indicated by p-value (*p<0.05, **p<0.01, ****p<0.0001), and ns denotes not significant.

The mean fluorescence intensity of CFSE was measured by flow cytometry to evaluate the proliferation ability of CAR-T cells as described previously (7). In the absence of target cells, all the tested groups demonstrated identical proliferation rates except shD-NKG2D-CAR-T cells. ShD-NKG2D-CAR-T cells demonstrated a significant decrease in MFI of CFSE after five days with and without co-culture, implying that HDAC11 gene interference improves CAR-T cell proliferation (**Figure 4A**). It has been suggested previously that inhibition of the histone deacetylation process can decrease T cell exhaustion. Additionally, treatment of T cells with HDAC inhibitors led to the differentiation towards a T central memory phenotype (T_CM_) (18, 19). So we analyzed the exhaustion and differentiation phenomenon. The cells were co-cultured with PC-3 and were analyzed for PD-1 and TIM3 surface expression via flow cytometry. shD-NKG2D-CAR-T cells demonstrated decreased PD-1 and TIM3 double positive cells as compared to the NKG2D-CAR-T group at different time points (**Figure 4B**). Furthermore, the proportion of T central memory cells (T_CM_) was significantly higher in shD-NKG2D-CAR-T (11.5% ± 0.707 vs. 20.3% ± 0.707) as compared to NKG2D-CAR-T group (**Figure 4C**). It means that HDAC11 gene interference in CAR-T cells can promote their differentiation into T_CM_ which represents a superior T cell phenotype with longer persistence and is beneficial to durable therapeutic effect for CAR-T cells. In consideration of prior findings (20, 21), we hypothesized that HDAC11 may exert regulatory effects on CAR-T cell functions by influencing Eomes expression. Supporting this hypothesis, the shD-NKG2D-CAR-T cells displayed significantly higher proportions of Eomes positive cells (15.9% ± 0.77 vs. 27.1% ± 1.27) compared to NKG2D-CAR-T cells (**Figure 4D**). The collective findings from our *in vitro* data demonstrate that HDAC11 plays a pivotal role in regulating CAR-T cell exhaustion and differentiation, which are closely associated with the expression levels of the Eomes transcription factor.

### 3.5 HDAC11 downregulation improves CAR-T therapy efficiency against prostate tumor xenografts

**Figure 5:**
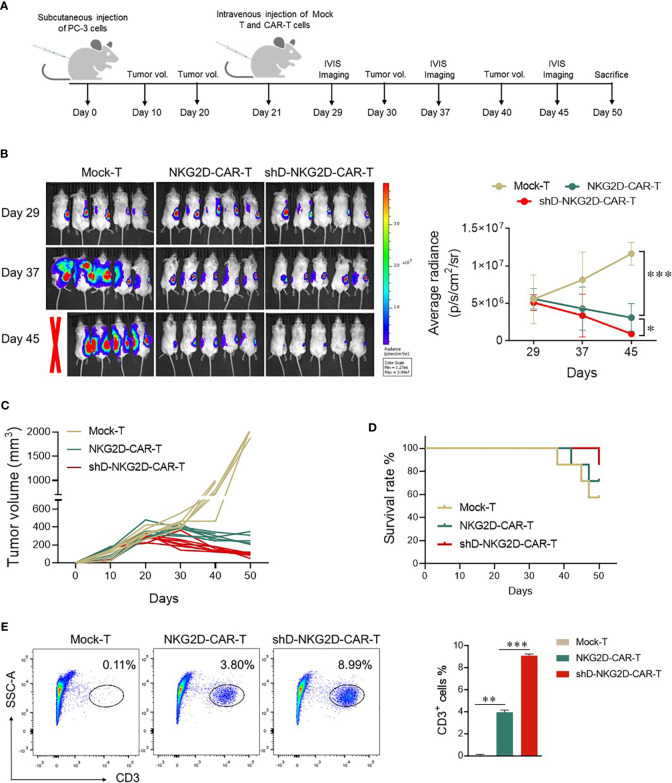
shD-NKG2D-CAR-T cells inhibited the growth of PC-3 subcutaneous xenografts. **(A)** Schematic outline of *in vivo* experimental design. NOD/SCID/γc-/- (NSG) mice were subcutaneously injected with 5×10^6^ PC-3-luciferase cells. On day 21, the tumor-bearing mice were randomized and treated with NKG2D-CAR-T (1×10^7^, n=7), shD-NKG2D-CAR-T cells (1×10^7^, n=7), or Mock-T cells (1×10^7^, n=7) as a control. **(B)** Tumor burden was monitored using bioluminescence intensity from the IVIS imaging system at various time points. Data is presented from n=5/group. The left panel shows representative IVIS imaging and right panel illustrates average radiance (p/s). **(C)** Tumor volume was assessed every 10 days using calipers. Each line on the graph represents the tumor progression for an individual mouse. **(D)** The overall survival data until 50 days after treatment for each group was depicted through a Kaplan-Meier survival curve. **(E)** On day 50, mice were sacrificed and tumor tissues were extracted. After preparing a single cell suspension, cells were stained with BV421-conjugated anti-human CD3 antibody, and flow cytometry was performed. Statistical significance was determined using one-way ANOVA followed by Tukey’s test and indicated by p-value (*p<0.05, **p<0.01, ***p<0.001).

The impact of HDAC11 downregulation on the efficacy of CAR-T cell therapy against prostate tumor xenografts was investigated as detailed in **Figure 5A**. Subcutaneous injection of luciferase-expressing transgenic PC-3 cells into mice set the stage for the experiments. Following the random allocation of mice in Mock-T, NKG2D-CAR-T, and shD-NKG2D-CAR-T groups, the corresponding treatments were administered. IVIS imaging sessions were conducted on day 29, 35, and 47 post-tumor inoculation.

Mice administered Mock-T cells exhibited a time-dependent increase in average radiance, paralleled by larger tumor volumes at the study’s end. In contrast, the shD-NKG2D-CAR-T group, as compared to the Mock-T and NKG2D-CAR-T cohort, demonstrated reduced average radiance and tumor volumes (**Figures 5B, C**). Moreover, the survival rate of the shD-NKG2D-CAR-T group was also better than that of the NKG2D-CAR-T cohort (**Figure 5D**), although one mouse in shD-NKG2D-CAR-T group suffered accidental death. On day 50, mice were sacrificed and tumors were excised, the result showed that less tumor size in shD-NKG2D-CAR-T-treated group was observed than wild-type CAR-T-treated mice (**Supplementary Figure S5**). Subsequent analysis of excised tumors from the experimental groups, focusing on the number of tumor-infiltrating T cells, was performed. Flow cytometry data indicated that HDAC11 downregulation notably enhanced the infiltration of CAR-T cells within the tumor microenvironment of prostate tumor mice xenografts (**Figure 5E**).

### 3.6 HDAC11 downregulation increases the functional persistence of circulating CAR-T cells

**Figure 6:**
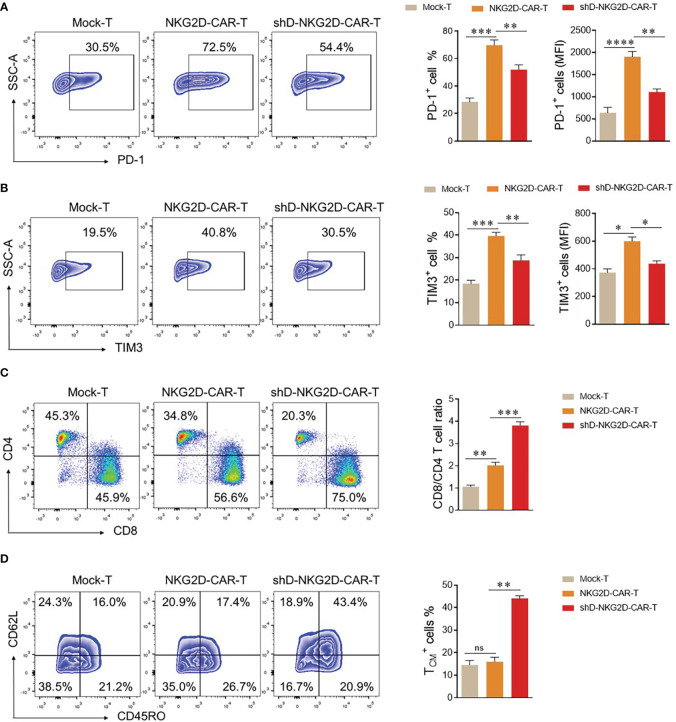
shD-NKG2D-CAR-T cells increase the less differentiated T cell population *in vivo***. (A–D)** On day 50, blood samples were collected from mice (n=3/group) via the retro-orbital method in EDTA-containing eppendorf tubes. Red blood cell lysis buffer was added to remove RBCs, and cells were stained with respective antibodies. **(A, B)** For exhaustion marker profiling, cells were stained with PE-conjugated anti-human PD-1 and APC-conjugated anti-human TIM3 antibodies. Flow cytometry analysis revealed PD-1 **(A)** and TIM3 **(B)** expression. **(C)** For T cell subset evaluation, cells were stained with BV421-conjugated anti-human CD3, APC-conjugated anti-human CD4, and PE-conjugated anti-human CD8 antibodies. **(D)** For T cell differentiation, cells were stained with BV421-conjugated anti-human CD3, PE-conjugated anti-human CD62L, and APC/Cy7-conjugated anti-human CD45RO antibodies and subjected to flow cytometry. Forward vs. side scatter plot gating strategy was used in all experiments. CD62L and CD45RO double fluorescence were detected under CD3 gating. FACS files are presented in the left panel while corresponding column charts are on the right. Statistical significance was determined using one-way ANOVA followed by Tukey’s test. Statistical significance is indicated by p-value (*p<0.05, **p<0.01, ***p<0.001, ****p<0.0001), and ns denotes not significant.

The CAR-T cells are detectable in the blood of patients receiving transduced cells. However, it is crucial to differentiate between their mere presence and functional persistence, as the latter is essential for a durable immunotherapeutic response (22). To validate the functional persistence of circulating CAR-T cells, blood was drawn from tumor-bearing mice of experimental cohorts at the end of the experiment. The CAR-T cells were isolated from the blood and subjected to flow cytometry analysis. We quantified the expression levels of co-inhibitory receptors PD-1 and TIM3 within the experimental cohorts, recognizing that sustained antigenic stimulation frequently culminates in cellular exhaustion. The flow cytometry analysis delineated a discernible reduction in the proportions of PD-1 and TIM3 in the HDAC11-deficient CAR-T cell population when compared with their conventional CAR-T cell counterparts (**Figures 6A, B**). Additionally, shD-NKG2D-CAR-T cells exhibited an elevated percentage of CD8^+^ cells relative to their wild-type equivalents (**Figure 6C**). In subsequent analyses, we delved into the quantification of T_CM_ cells within the experimental cohorts. In line with the *in vitro* findings, the HDAC11-deficient CAR-T cells displayed augmented formation of memory cells *in vivo*, as illustrated in **Figure 6D**. Overall, the data signifies that the absence of HDAC11 signaling in CAR-T cells correlates with reduced exhaustion, imparting these cells with the ability to afford enduring anti-tumor control upon antigen rechallenge.

## 4 Discussion

The NKG2D receptor has gained substantial attention in immunotherapy-based strategies to effectively eradicate tumors in experimental settings. One of the major advantages they offer is excellent ligand expression selectivity on tumor cells. NKG2D-based CAR-T cell therapies have been demonstrated to be safe for ligand-negative cells, making them excellent targets in cancer immunotherapies (6, 7, 23, 24). *In vitro* experiments demonstrated that pretreating ovarian cancer cells with valproic acid (an HDAC inhibitor) resulted in an elevated expression of NKG2D ligands, consequently enhancing the efficacy of NKG2D-CAR-T cell therapy (15). Another study further validated the analogous mechanism *in vitro* and *in vivo* settings (16). The study showcased that administering valproic acid to mice heightened the effectiveness of CAR-T cell therapy against acute myeloid leukemia. The findings indicated that valproic acid augmented the expression of NKG2D ligands in target cells, thereby enhancing CAR-T cell activation and cytokine release (16). Notably, both of the aforementioned studies harnessed HDAC inhibition in target cells to improve CAR-T cell therapy efficacy. In the present study, we have introduced an innovative approach where interference of the HDAC11 gene in CAR-transduced T cells could significantly enhance the potency of NKG2D-based CAR-T cell therapy. We have specifically downregulated HDAC11 gene in CAR-T cells by incorporating HDAC11 shRNA in NKG2D-chimeric antigen receptor. To the best of our knowledge our study is the first to report that HDAC11 downregulation in CAR-T cells can improve their immunotherapeutic outcome.

Enhancing the efficacy of CAR-T cell therapy through RNAi has been done by our group against pancreatic and prostate (cancer) previously (7, 25). While targeting pancreatic cancer with NKG2D-targeting CAR-T cells, we demonstrated that downregulation of 4.1R could enhance CAR-T therapy efficacy. An improved therapeutic outcome was observed in 4.1R-silencing CAR-T cells (7). Additionally, in prostate cancer, CAR-T cells were empowered by downregulating the cholesterol esterification enzyme ACAT1 which was also achieved through shRNA technology (25). In current study, we designed four shRNA sequences for HDAC11 and chose the one giving the best interference efficiency. The best shRNA was then inserted into second generation NKG2D-CAR vector, and successful downregulation was validated via western blot. shD-NKG2D-CAR-T cells displayed better anti-tumor efficacy against PC-3 and DU-145 cancer cells. Although the cytotoxicity analysis against PC-3 and DU-154 cells were done in a separate set of experiments, the differential cytotoxicity was observed which might be due to the different NKG2DL expression level on the two target cells. HDAC11 downregulation does not affect T cell functions in resting conditions, but a negative correlation between HDAC11 expression and T cell activation has been observed previously (20). Our results are in agreement with this study, where shD-NKG2D-CAR-T displayed enhanced CD69 and CD107a expression as compared with NKG2D-CAR-T cells as a measure of T cell activation and degranulation capacity (26, 27).

HDAC11 downregulation also led to an increased cytokine and GzmB expression in CAR-T cells. Increased cytolytic activity in shD-NKG2D-CAR-T cells might correspond to increased activation and IFN-γ production in response to stimulation by cancer cells. HDAC11 downregulation is responsible for increased proliferation in T lymphocytes, as demonstrated by greater ki67 expression previously (28). In the current study, HDAC11 knockdown in NKG2D-CAR-T cells led to increased T cell proliferation, reduced exhaustion, and a less differentiated CAR-T cell phenotype upon exposure to tumor antigens. This was ascribed to the involvement of transcription factor Eomes (**Figure 4**), which are controlled by HDAC11 and play a crucial role in modulating T cell functions (29). After administration, CAR-T cells may undergo exhaustion due to various factors, including prolonged exposure to the tumor microenvironment, persistent antigen stimulation, and immunosuppressive signals (30). Besides the factors mentioned, histone deacetylation may contribute to T cell exhaustion phenotypes (31). shD-NKG2D-CAR-T cells demonstrated less proportions of PD-1 and TIM3 double positive cells *in vivo*, which can be attributed to decreased histone deacetylation in these cells. Subsequently, *in vitro* cytotoxicity ability of shD-NKG2D-CAR-T cells was authenticated in an *in vivo* setting, where shD-NKG2D-CAR-T cells demonstrated superior anti-tumor efficacy in a prostate cancer xenograft model. Futhermore, the mice receiving shD-NKG2D-CAR-T demonstrated decrease exhaustion and increased memory formation at the end of study. Overall, HDAC11 downregulation offers an excellent opportunity to augment the effectiveness of immune-based therapies. More studies are needed to unearth the role of epigenetic mechanisms governing CAR-T cell functions. These results collectively underscore the pivotal role of HDAC11 in shaping CAR-T cell functionality and present a promising avenue for enhancing the immunotherapeutic response against prostate cancer. The observed improvements in CAR-T cell attributes open avenues for translational research, offering novel strategies to optimize CAR-T cell therapy for improved clinical outcomes.

## 5 Conclusions

The findings suggest that HDAC11 knockdown holds promise as a strategy to enhance the therapeutic impact of CAR-T cells, with potential implications for improving patient outcomes in prostate cancer treatment. The idea of enhancing CAR-T cell function by manipulating HDAC11 expression will provide valuable insights for the development of optimized immunotherapeutic strategies in the future.

## Data availability statement

The original contributions presented in the study are included in the article/**Supplementary Material**. Further inquiries can be directed to the corresponding author.

## Ethics statement

The animal study was approved by Institutional Animal Care and Use Committee of East China Normal University. The study was conducted in accordance with the local legislation and institutional requirements.

## Author contributions

HZ: Conceptualization, Data curation, Methodology, Writing – review & editing. JY: Data curation, Formal analysis, Methodology, Writing – review & editing. IA: Data curation, Formal analysis, Software, Validation, Writing – original draft, Writing – review & editing. MF: Validation, Writing – original draft, Writing – review & editing. WJ: Conceptualization, Funding acquisition, Investigation, Project administration, Resources, Supervision, Validation, Visualization, Writing – review & editing.
