# Supplementary material for: shRNA-mediated gene silencing of HDAC11 empowers CAR-T cells against prostate cancer
Source: Front Immunol. 2024 May 21;15:1369406. doi: 10.3389/fimmu.2024.1369406 (PMC11148219; doi:10.3389/fimmu.2024.1369406)
Supplement: Supplementary file 1 [file DataSheet_1.docx]

**Supplementary Material:**

**Table S1** HDAC11-shRNA oligonucleotide sequences.


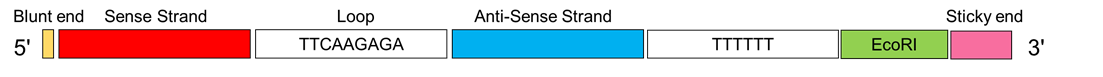


| **Target Name** |  | **shRNA sequence (5'-3')** |
| --- | --- | --- |
| HDAC11-shRNA-A | F | TGGAGAAGCTGCATCCCTTTGATTCAAGAGATCAAAGGGATGCAGCTTCTCCTTTTTTGAATTCC |
|  | R | TCGAGGAATTCAAAAAAGGAGAAGCTGCATCCCTTTGATCTCTTGAATCAAAGGGATGCAGCTTCTCCA |
| HDAC11-shRNA-B | F | TGCACACGAGGCGCTATCTTAATTCAAGAGATTAAGATAGCGCCTCGTGTGCTTTTTTGAATTCC |
|  | R | TCGAGGAATTCAAAAAAGCACACGAGGCGCTATCTTAATCTCTTGAATTAAGATAGCGCCTCGTGTGCA |
| HDAC11-shRNA-C | F | TGGCTACCATCATTGATCTTGATTCAAGAGATCAAGATCAATGATGGTAGCCTTTTTTGAATTCC |
|  | R | TCGAGGAATTCAAAAAAGGCTACCATCATTGATCTTGATCTCTTGAATCAAGATCAATGATGGTAGCCA |
| HDAC11-shRNA-D | F | TGGATGATGAGTACCTGGATAATTCAAGAGATTATCCAGGTACTCATCATCCTTTTTTGAATTCC |
|  | R | TCGAGGAATTCAAAAAAGGATGATGAGTACCTGGATAATCTCTTGAATTATCCAGGTACTCATCATCCA |
| HDAC11-shRNA-NC | F | TGGATCCATAGTCGTGGTAATCTTCAAGAGAGATTACCACGACTATGGATCCTTTTTTGAATTCC |
|  | R | TCGAGGAATTCAAAAAAAGGATCCATAGTCGTGGTAATCTCTCTTGAAGATTACCACGACTATGGATCCA |


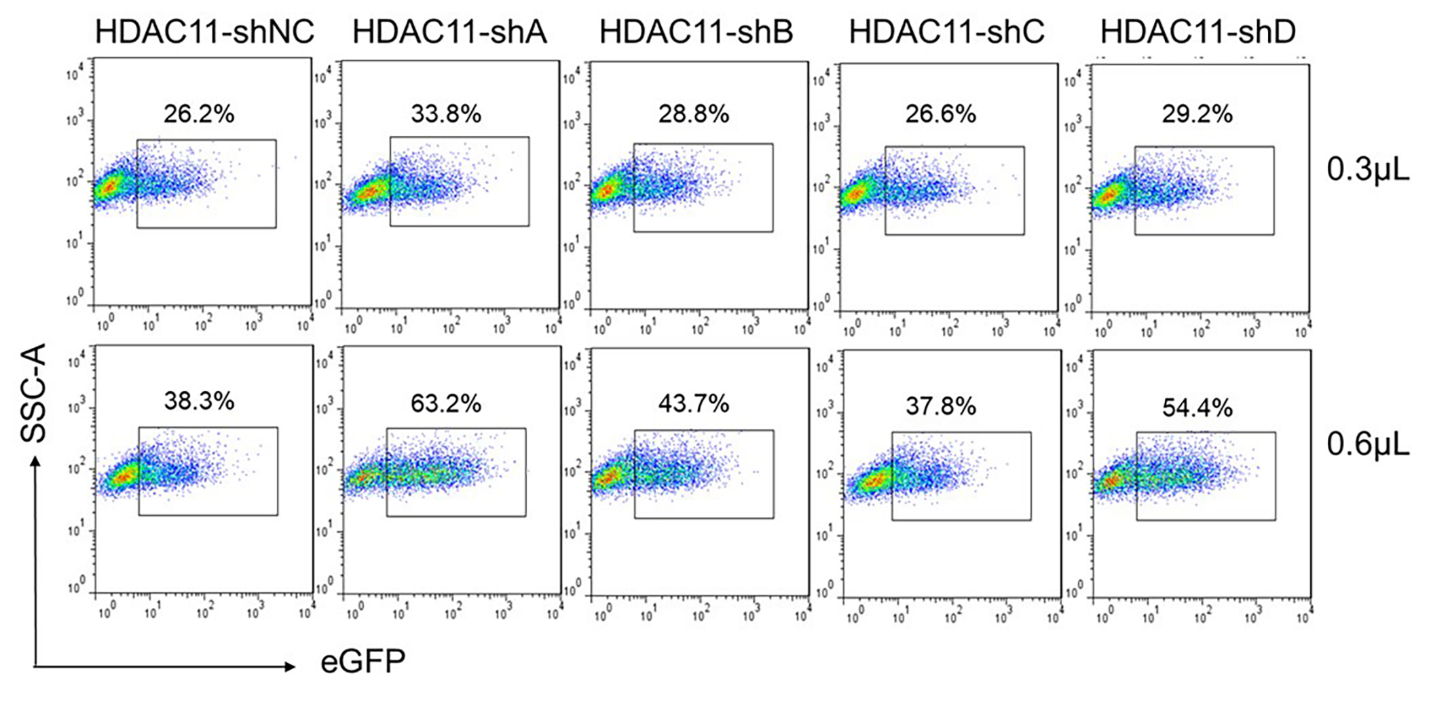


**Figure S1** Transduction efficiency of HDAC11-sh (A, B, C, D and NC) lentiviruses in HEK293T cells. 2×10^5^ HEK293T cells were seeded in a 24-well plate and infected with two different concentrations of lentiviruses from each group. After incubation for 48 hours, cells were subjected to flow cytometry for eGFP positive rate analysis. A forward vs. scatter plot gating strategy was employed, and a positive rate was measured.


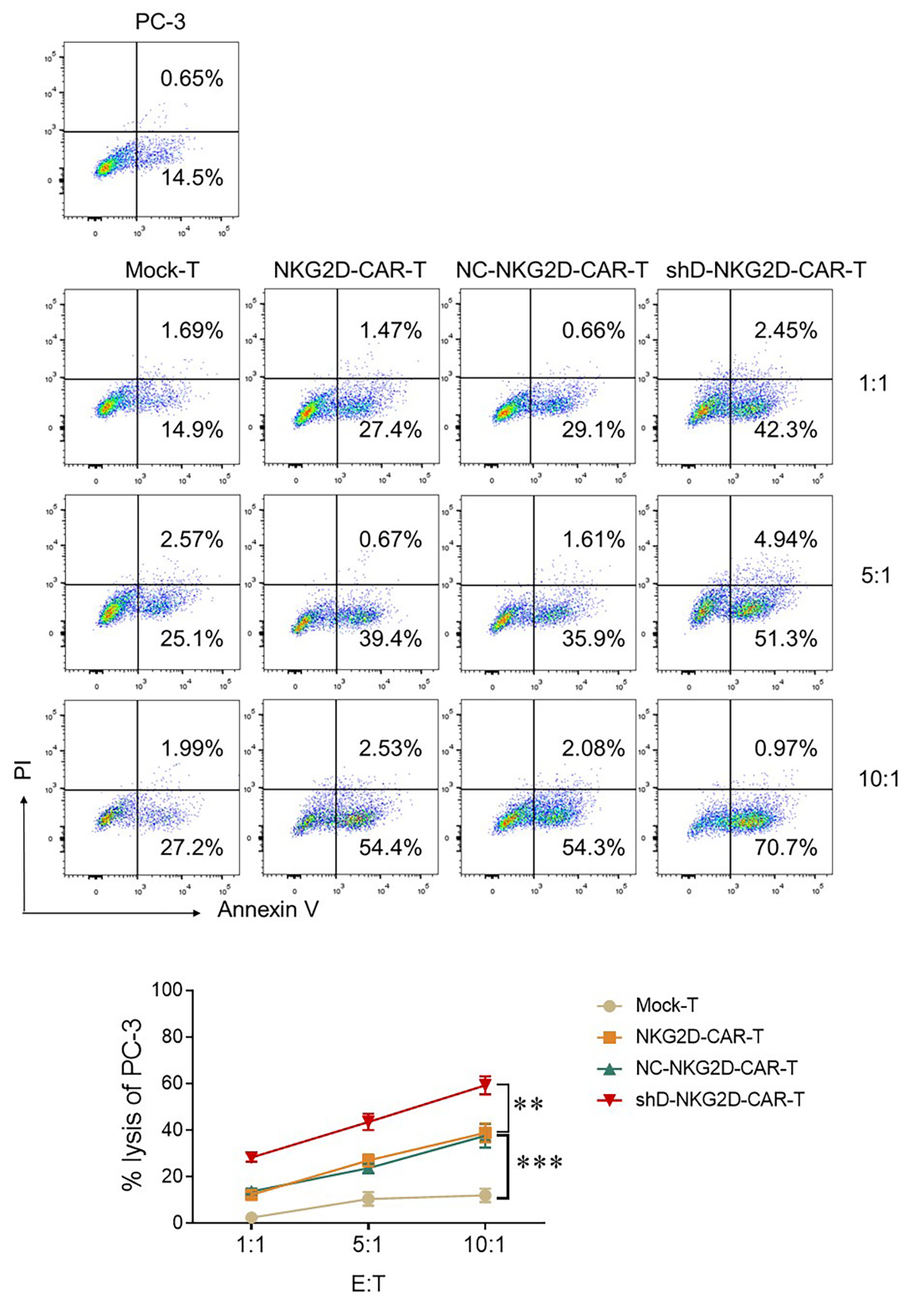


**Figure S2** HDAC11 downregulation enhances the cytotoxic ability of NKG2D-CAR-T cells. PC-3 target cells were labeled with CFSE and co-cultured with Mock-T, NKG2D-CAR-T, shNC-NKG2D-CAR-T, or shD-NKG2D-CAR-T cells at various Effector to target ratios in a low adherence 96-well plate. After 12 hours, cells were collected and stained with Annexin V/ PI and immediately subjected to flow cytometry. Annexin V^+^ and PI^+^ cells were detected within CFSE-labeled target cells, and the percent lysis was quantified. Representative FACS files are presented in the upper panel, while the below panel presents the percent lysis in a line plot. Statistical analysis was conducted using a two-way ANOVA multiple comparison test, and significance levels were determined by p-value (**p<0.01, ***p<00.1), and ns denotes not significant.


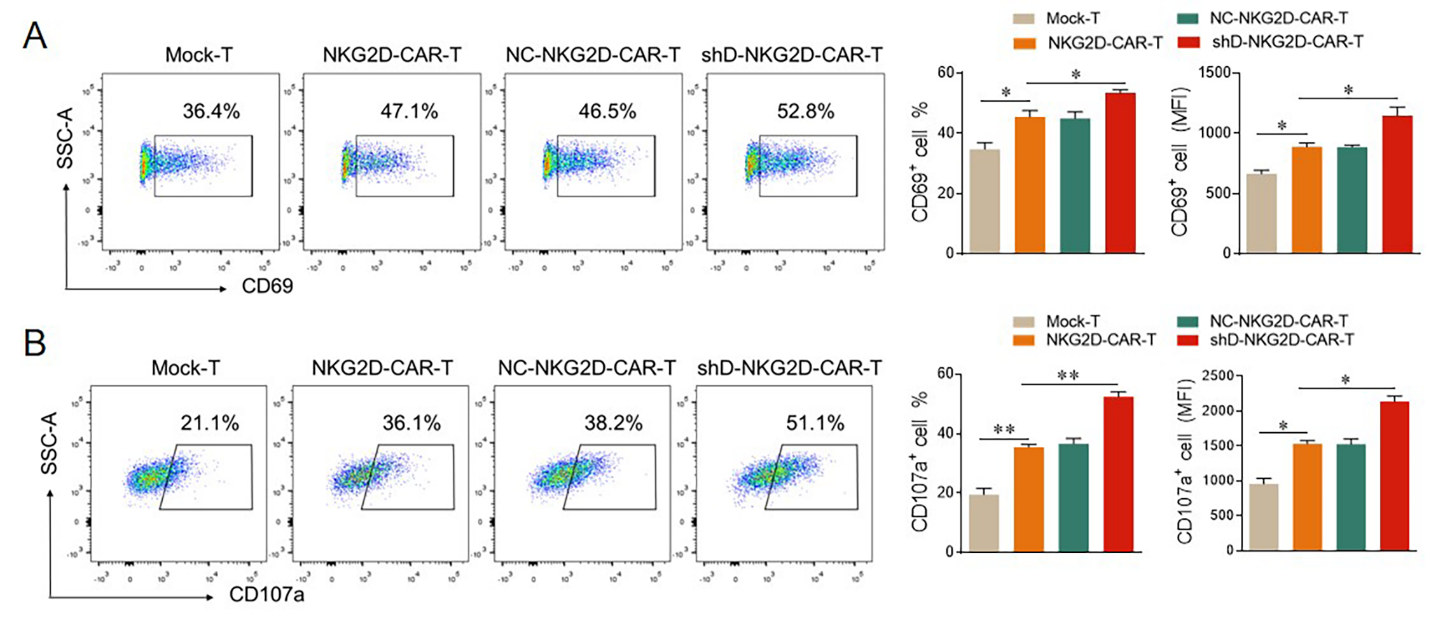


**Figure S3** HDAC11 downregulation enhances the activation and degranulation in NKG2D-CAR-T cells when co-cultured with DU-145 cells. Effector cells were co-cultured with DU-145 target cells in a 48-well plate. After 12 hours, cells were stained with APC-conjugated anti-human CD69 and PE/Cy7-conjugated anti-human CD107a antibodies to detect **(A)** CD69 and **(B)** CD107a expression via flow cytometry.


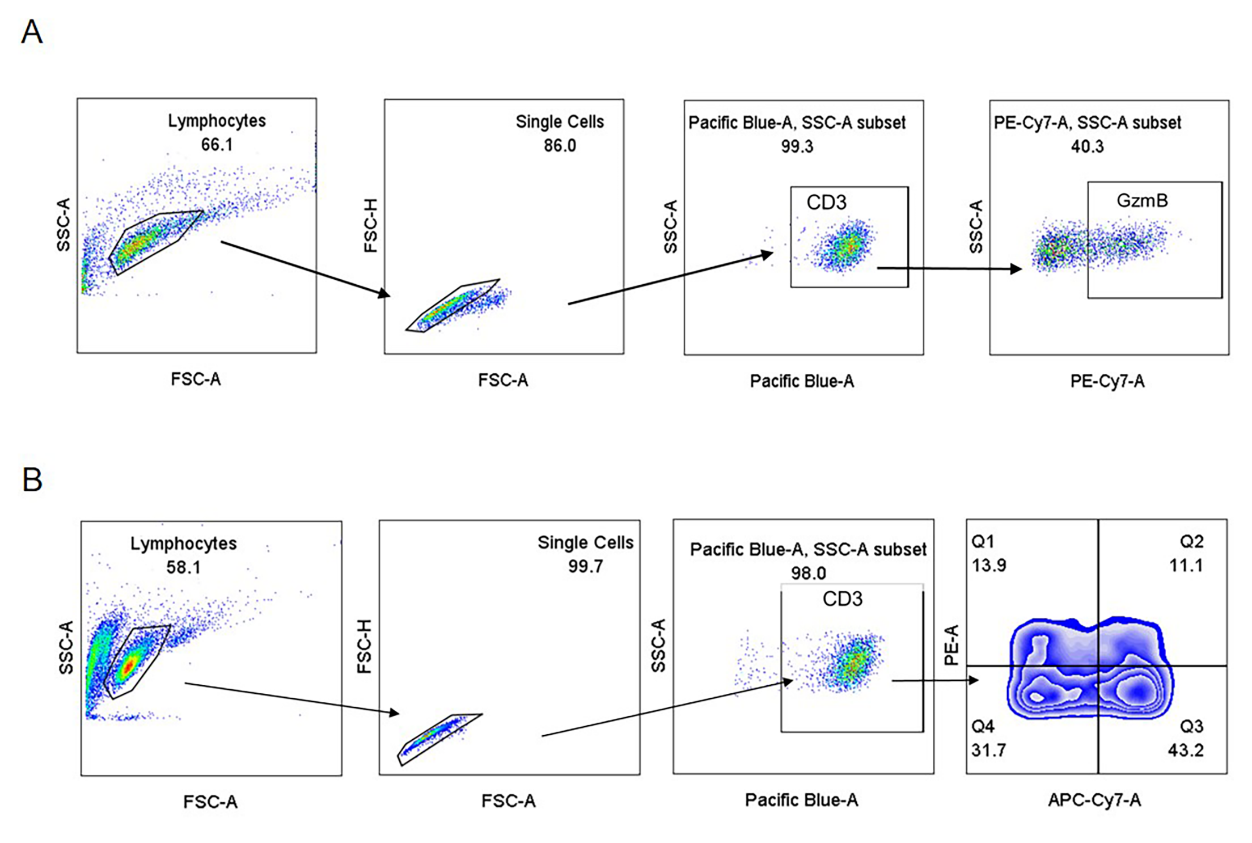


**Figure S4** Gating strategy for flow cytometry analysis. **(A)** To detect single fluorescence, cells were initially stained with BV421-conjugated anti-human CD3 antibody. Subsequently, specific marker antibodies (CD69, CD107a, GzmB, IFN-γ, PD-1, TIM3, or Eomes) were added as described in their respective experiments. The gating strategy involved using forward vs. side scatter to identify CD3^+^ cells, and under these cells, single fluorescence was detected based on the specific marker antibodies. **(B)** For dual-fluorescence detection, cells were stained with BV421-conjugated antihuman CD3 antibody and with (CD4 and CD8, or CD62L and CD45RA) antibodies based on the experimental requirement. The gating strategy utilized forward vs. side scatter to identify CD3^+^ cells, and under these cells, double-fluorescence was detected in one scatter dot or zebra plot.


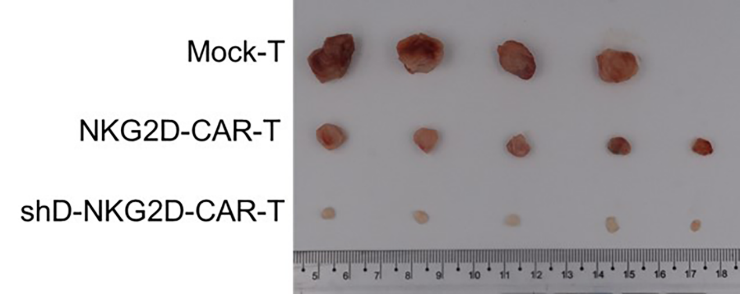


**Figure S5** Tumor size. On day 50, mice were sacrificed and tumors were extracted from PC-3 xenografts treated with Mock-T, NKG2D-CAR-T, and shD-NKG2D-CAR-T cells, and a digital image was taken out.
